# Dr. Walter M. Litchfield

**Published:** 1916-03

**Authors:** 


**Dr. Walter M. Litchfield,** Buffalo 1886, died at his home in Salamanca Feb. 9, aged 53. He came to Salamanca from Independence, Kas., six years ago. He was an oil producer.

